# Effects of task-oriented treadmill training applied high-intensity interval training on walking ability in patients with chronic stroke: A randomized controlled trial with short-term follow-up

**DOI:** 10.1097/MD.0000000000044444

**Published:** 2025-09-19

**Authors:** Kyu-Ryeong Kim, Ye-Ji Kim, Myoung-Kwon Kim

**Affiliations:** a Department of Physical Therapy, Graduate School, Daegu University, Gyeongsan, Gyeongbuk, Republic of Korea; b Department of Physical Therapy, College of Rehabilitation Sciences, Daegu University, Gyeongsan, Gyeongbuk, Republic of Korea.

**Keywords:** high-intensity interval training, stroke, stroke recovery, walking

## Abstract

**Background:**

This study aimed to determine the effect of task-oriented treadmill training with high-intensity interval training compared to high-intensity interval treadmill training on the walking ability of patients with chronic stroke.

**Methods:**

Data from 34 chronic stroke patients who were randomly assigned to experimental (17) and control (17) groups and completed the study were analyzed. The experimental group underwent task-oriented treadmill training with high-intensity interval training, and the control group received high-intensity interval treadmill training for 30 minutes per session, 3 times a week, over a period of 4 weeks. Statistical significance was confirmed through a 2-factor repeated measures analysis of variance.

**Results:**

There was an interaction effect between time and group for 10-Meter Walk test (10MWT), functional gait assessment (FGA), 6-Minute Walk test (6MWT), and timed up and go (TUG) (*P* < .05). post hoc tests revealed that 10MWT, FGA, and 6MWT showed significantly greater improvements in the e.g. compared to the CG (*P* < .025), and in the intragroup comparison, both e.g. and CG showed significant improvements over time (*P* < .017). The TUG showed no significant difference in comparison between groups (*P* > .025), and in comparison within groups, both e.g. and CG showed a significant decrease over time (*P* < .017).

**Conclusion:**

Clinically, despite being a chronic stroke patient, repetitive and meaningful task training combined with high-intensity training is thought to be helpful in improving walking ability and returning to the community.

## 1. Introduction

### 1.1. Necessity of study

According to the World Health Organization, a stroke is a condition where symptoms last for over 24 hours due to vascular causes or a local or general impairment of brain function, potentially resulting in death.^[1]^ Stroke is a significant cause of death worldwide, with 133 million stroke cases reported globally.^[2]^

Stroke patients are affected by the extent of brain lesions and related areas, with motor impairment being a typical characteristic.^[3,4]^ Delays in motor ability recovery can result in asymmetric weight bearing on the paralyzed and non-paralyzed sides, reduced weight-shifting ability, and difficulty in maintaining an independent standing position.^[5]^ This can impede normal walking and limit activities of daily living,^[6,7]^ as well as restrict community participation.^[8]^

In particular, the decrease in walking ability is one of the most common functional limitations of stroke patients,^[6]^ and the recovery of walking ability is considered the most important aspect in the rehabilitation of stroke patients.^[9,10]^

After a stroke, 50% of early stroke patients cannot walk, 12% can walk only with assistance, and only 37% can walk independently.^[11]^ Even after 11 weeks of continuous rehabilitation, 18% of patients are unable to walk, 11% require assistance when walking, and only approximately 50% can walk independently.^[11]^ Among patients who experienced physical disability due to stroke, 60 to 80% are able to walk independently.^[12]^ However, a significant number of patients exhibit a slow walking speed of 0.38 to 0.80 m/s, which hinders the demonstration of stable walking ability in the community.^[12]^

In addition, stroke patients have significantly decreased cardiopulmonary endurance, resulting in reduced walking endurance, limited participation in community activities, such as being unable to cross crosswalks quickly or walk independently in crowded places.^[8,13,14]^

A number of recent studies on treadmill training as an intervention to improve walking ability in stroke patients^[15–17]^ suggest that high-intensity treadmill training is more effective for walking than low-intensity or moderate-intensity treadmill training in stroke patients.^[18–20]^

However, treadmill training alone is not sufficient to change the walking ability of stroke patients in various walking environments.^[21]^ The walking environment in everyday life is constantly exposed to unexpected conditions, and stroke patients lack the ability to adapt to these environments compared to individuals without stroke.^[22]^ Adaptation to the external environment affects motor learning, depending on the similarity of training between the 2 environments.^[23]^ The more similar the situations in the training environment are to the real environment, the more effective the motor learning in the new environment becomes.^[23,24]^

Therefore, efficient walking for stroke patients involves task-oriented activities in a variety of real-world settings as part of as a strategic social adaptation treatment program.^[24–26]^ Studies show that obstacle-crossing task-oriented treadmill training for stroke patients can reduce the risk of falling and is effective in improving walking speed and balance ability.^[27–29]^

Previous studies have shown that high-intensity aerobic walking training is effective in improving balance ability, walking speed, and walking endurance. However, there is a lack of evidence regarding walking ability in challenging environments such as curved roads, obstacles, and unstable ground, which are common in community settings and relevant to daily life. Additionally, there is a scarcity of studies on conducting walking training within environmental constraints, incorporating obstacles, and determining their impact on learning.

### 1.2. Purpose of study

This study aims to investigate the effects and learning outcomes of high-intensity aerobic training on walking ability for chronic stroke patients. The training program combines daily life tasks with obstacle-crossing exercises.

## 2. Methods

### 2.1. Experimental subjects

Subjects were 34 patients diagnosed with stroke at the hospital. All participants were recruited from the inpatient ward of K Hospital in Korea, a rehabilitation hospital, and provided informed written consent before enrolling in the study. This study was approved by the Research Ethics Committee of Daegu University (1040621-202307-HR-058).

The inclusion criteria were as follows: post-stroke duration of at least 6 but not more than 24 months; a score on the Mini-Mental State Examination not lower than 24; ability to walk at least 10 meters with or without any walking aid; and walking speed < 0.8 m/s.

The exclusion criteria were as follow: visual and cognitive impairment; heart disease and circulatory system problem; and difficulty moving due to medical instability or pain in the lower extremities.

To determine the sample size in this study, a pilot study was performed with 6 subjects. The pilot study measured mainly 10WMT and 6-Minute Walk test (6MWT) data, referring to the study by Madhavan et al.^[30]^ And then, the G*power program 3.1.0 (G power program Version 3.1, Heinrich-Heine-University Düsseldorf, Düsseldorf, Germany) was used based on the pilot study data. Based on the power analysis presented by Cohen, an effect size of 0.5, a significance level of 0.05, and a power of 80% were used to calculate the required sample size, which resulted in a total of 34 subjects.^[31]^

### 2.2. Experimental procedure

#### 2.2.1. Experimental design

**Figure 1.**
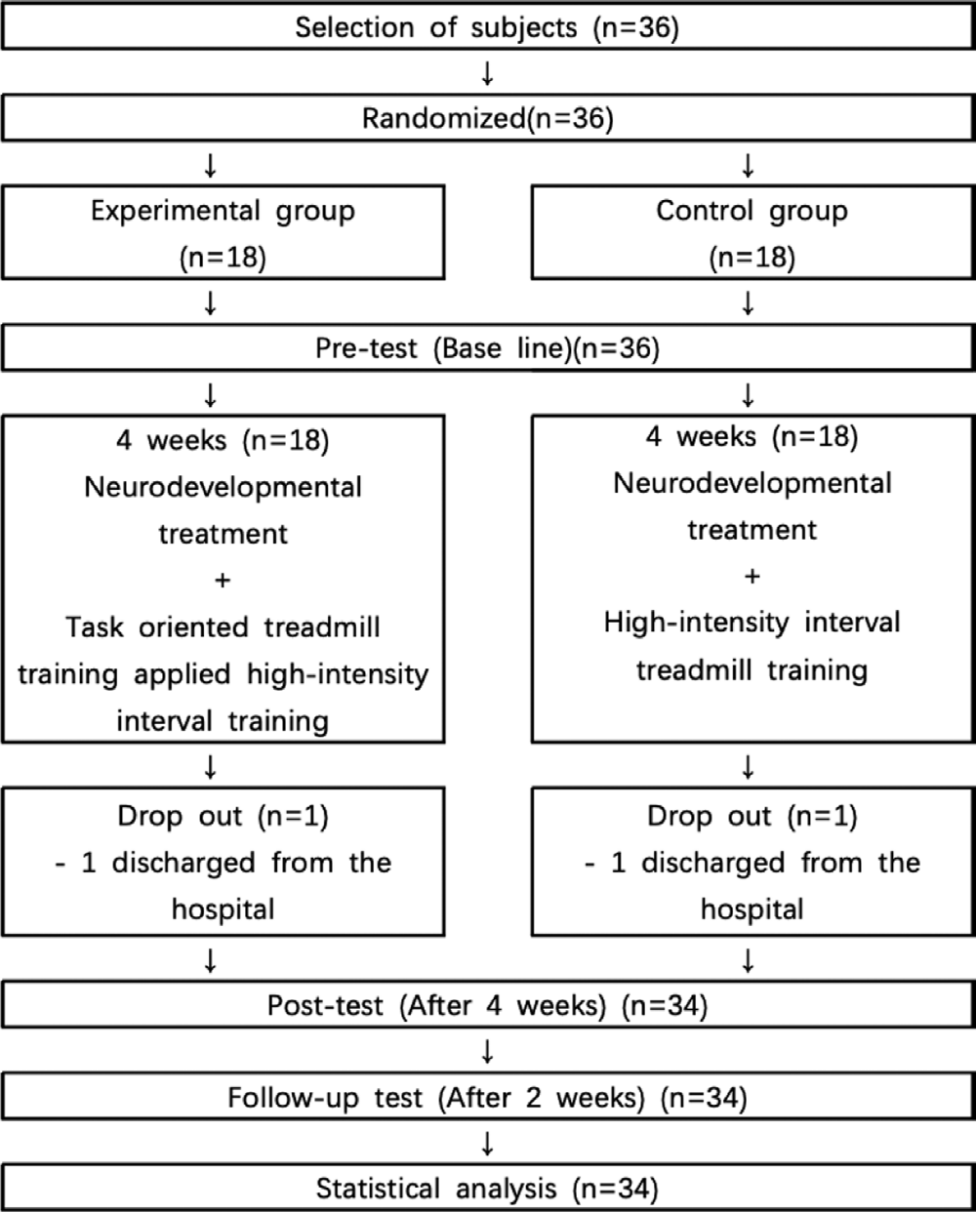
Study flow chart.

The purpose of the experiment was explained, and 36 patients who voluntarily agreed to the selection criteria were selected. They were then randomly assigned to the experimental group (n = 18) and the control group (n = 18). The random allocation was performed using a random allocation program (Random Allocation Software 2.0, University of Medical Sciences, Iran, 2008) to minimize selection bias. Random allocation was performed by a graduate student with no clinical involvement in the trial, and for allocation concealment, sealed envelopes sequentially numbered and opaque were used. The envelopes were kept in a location distinct from the assessment place and were not available to the assessor or the data analyst. The envelope was signed, dated, and opened by the allocation examiner immediately before the intervention, and only in the absence of the assessor and the data analyst. The study investigated the general characteristics of the subjects, including gender, age, height, weight, and type of injury, and conducted a pretest. After 4 weeks of training, a post-test was performed with the same content as the pretest. Before the post-test was completed, one participant from both the experimental and control groups was discharged. Subsequently, a post-test was administered to a total of 34 subjects, with 17 in the experimental group and 17 in the control group. A follow-up test was performed 2 weeks after the post-test to assess the learning effect (Fig. 1).

The experimental group underwent task-oriented interval treadmill training with high-intensity sessions lasting 30 minutes, 3 times a week, for a total of 12 sessions over 4 weeks. In contrast, the control group engaged in high-intensity interval treadmill training for 30 minutes, 3 times a week, totaling 12 sessions over 4 weeks. Both the experimental group and the control group also underwent NeuroDevelopment Treatment (NDT) treatment for 30 minutes, 5 times a week, for 4 weeks. NDT consists of neurodevelopmental therapy, muscle-strengthening exercise, joint range of motion exercise, balance training, and gait training.

**Figure 2.**
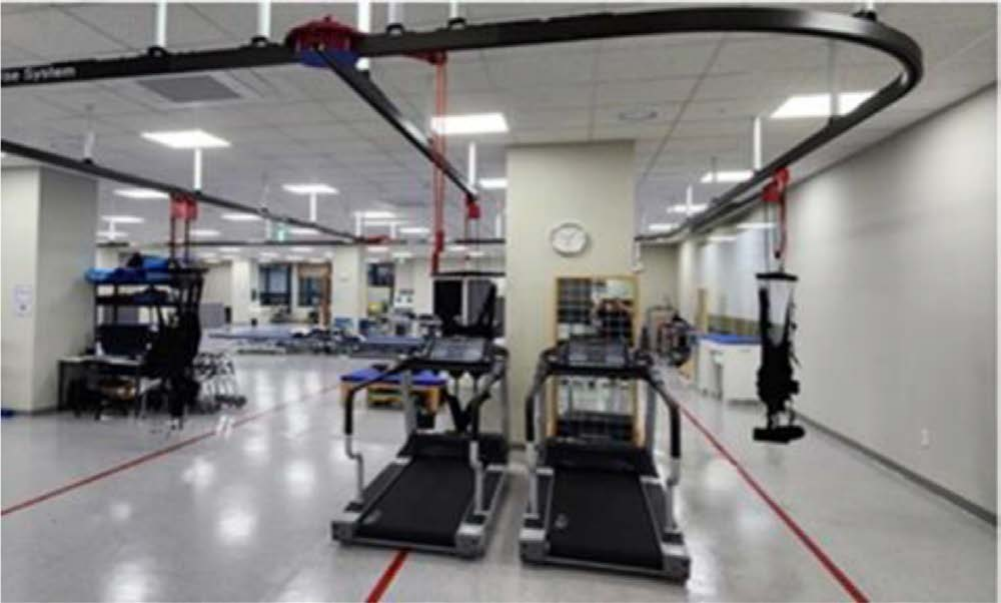
Suspension device (walking exercise system) and treadmill. Suspension device and treadmill were used for safety of the participants.

In order to prevent the subjects from falling during high-intensity interval treadmill training in both the experimental and control groups, a harness was installed and utilized on the treadmill^[32]^ (Fig. 2). In this study, treadmills for rehabilitation treatment (Fitex 6080, Fitex, Korea) were used for experiments, and walking exercise systems (Walking Exercise System, NEO-TECH, Korea) were used as suspension devices (Fig. 2).

For the safety of the subject, a protective vest was worn, safety bars were installed on the left and right sides of the treadmill, and a treadmill equipped with an automatic stop feature was selected to halt the power when the subject moved away from the treadmill. The experiment was conducted only under the supervision of a physical therapist to ensure a prompt response in case of a safety accident.

#### 2.2.2. Task-oriented treadmill training applied high-intensity interval training

**Figure 3.**
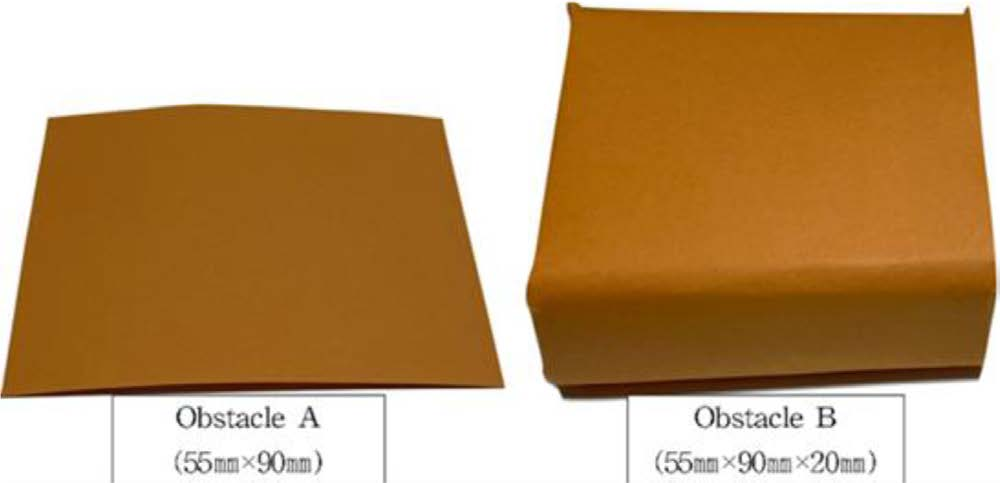
Obstacles. Obstacles used for task-oriented training obstacle A was 55 mm × 90 mm in size, and obstacle B was 55 mm × 90 mm × 20 mm in size.

**Figure 4.**
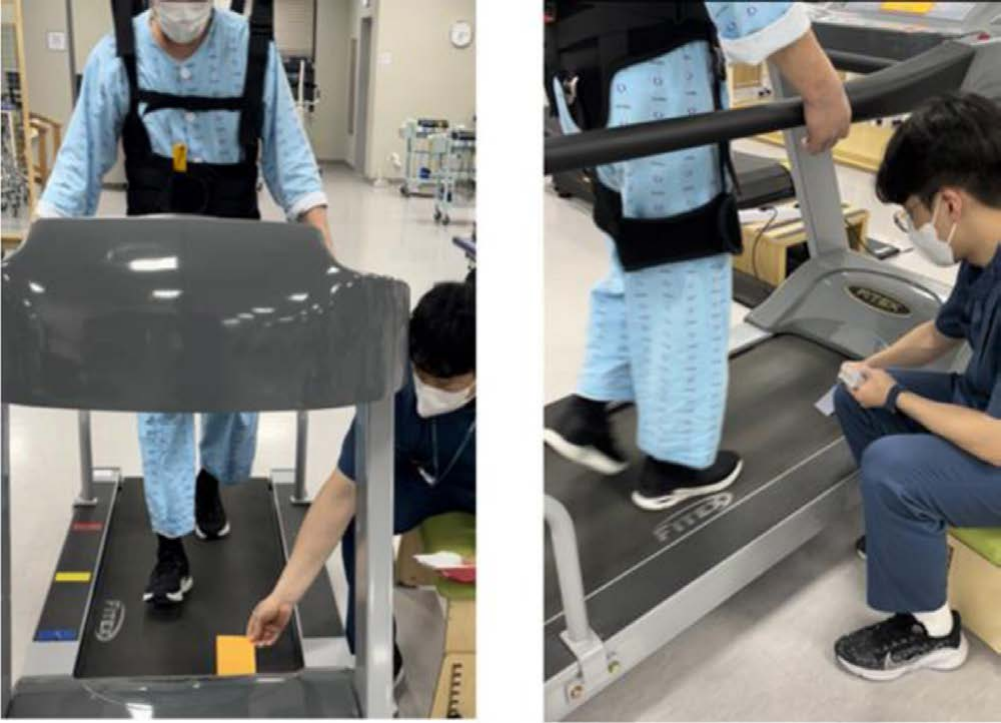
Task-oriented treadmill training applied high-intensity interval training. Task-oriented treadmill training applied high-intensity interval training applied obstacle A and obstacle B. For safety reasons, the subject wore a suspension device.

Task-oriented treadmill training involving high-intensity interval treadmill training was developed by adapting the approach proposed by Jeong and Koo^[27]^ and Amatachaya et al.^[33]^ Task-oriented training involves simulating obstacle situations commonly encountered at home or in the community. Manufacturing and using obstacles in 2 sizes (A: 55 mm × 90 mm, B: 55 mm × 90 mm × 20 mm) in different colors on the treadmill floor (Fig. 3). Obstacles were placed by the therapist directly onto the treadmill (Fig. 4), and the subject’s position was centered on the treadmill when crossing the obstacle. The order in which the obstacles are crossed is shown in Table 1. Task-oriented training was performed at high-intensity on a treadmill, and the description of high-intensity is as in 3) High-intensity interval treadmill training.

**Table 1:** Program of task-oriented treadmill.

Time	Direction to overcome obstacles (1)	Size of obstacles (2)
1 wk	Affected side	A: 55 mm × 90 mm
2 wk	Affected side	B: 55 mm × 90 mm × 20 mm
3 wk	Non affected side	Random
4 wk	Random	Random

#### 2.2.3. High-intensity interval treadmill training

**Figure 5.**
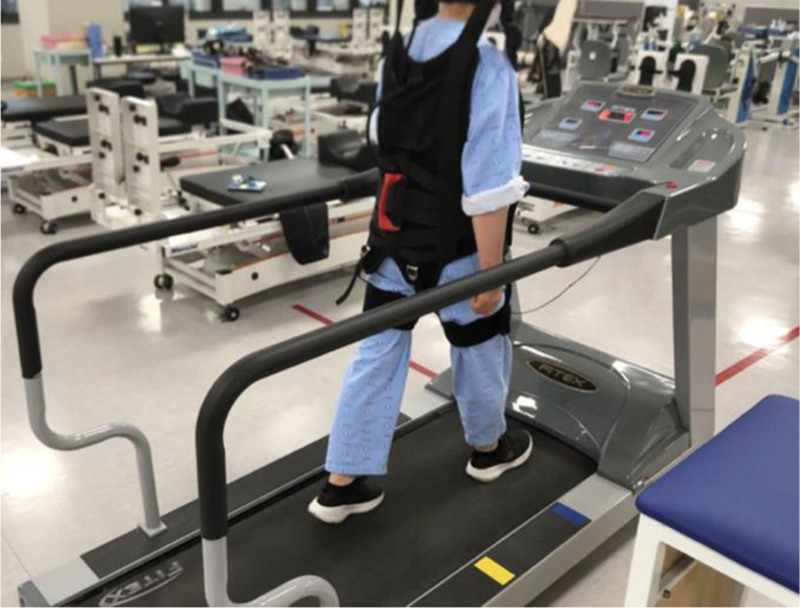
High-intensity interval treadmill training. High-intensity interval treadmill training was performed while wearing a suspension device for safety.

High-intensity interval treadmill training was conducted as part of a general treadmill training program (Fig. 5).

The target heart rate (Target HR) calculation method for determining the exercise intensity of high-intensity interval treadmill training for subjects involved using the Karvonen method. This method calculates the target heart rate by multiplying the percentage of the target exercise intensity by the heart rate reserve (maximum heart rate – resting heart rate) and then adding the resting heart rate (HR rest) at rest.^[34]^

The training session comprises a total of 30 minutes, with 5 minutes allocated for warm-up, 20 minutes for the main exercise to sustain the target heart rate, and 5 minutes for cool-down exercises.

This training protocol was modified to fit the situation based on research by Askim et al,^[35]^ Cleland and Madhavan,^[36]^, and Munari et al.^[18]^ When the subject reached the target heart rate corresponding to the high-intensity (70–80% exercise intensity), they selected a speed that could be maintained within the exercise intensity range and maintained the target heart rate for 2 minutes. After that, for an active break, the speed was lowered for 2 minutes to match the low-intensity (30–40% exercise intensity), and after 2 minutes, the speed was increased again to match the high intensity. During training, the subject adjusted to the speed and slightly increased it when the heart rate dropped.

The warm-up and cool-down exercises were conducted by slowing down to low intensity (30–40% exercise intensity). To maintain the target heart rate during training, a portable heart rate monitor device (Polar H7, Polar Electro) that can transmit heart rates to a mobile phone screen via Bluetooth was attached to the chest or arm.^[18]^ Once the target heart rate was set, the subject adjusted the treadmill speed to match the target heart rate. During training, if breathing-related symptoms were observed, chest pain was reported, the subject expressed difficulty, or pain occurred elsewhere, the training was immediately stopped. In addition, the training was verbally checked for continuity and immediately stopped when there was a request for suspension.^[37]^ Blood pressure, heart rate (HR), respiration rate, and oxygen saturation were monitored when the training was stopped. The following day, when reporting to the hospital doctor, a decision was made regarding whether to continue training.

### 2.3. Experimental instrumentation

#### 2.3.1. Timed up and go test

The timed up and go (TUG) can quickly evaluate basic motor performance and dynamic balance abilities.^[38]^ The TUG test measures the time taken to stand up from a chair without armrests, walk a distance of 3 meters, return to sit on the chair, and then sit back down at the therapist’s verbal command “Start..” Measurements were taken 3 times in total, and the average value was recorded. TUG is an assessment with very high reliability for stroke patients, and the test-retest reliability is ICC = .97.^[39]^

#### 2.3.2. Functional gait assessment

The functional gait assessment (FGA) is used to assess postural stability while walking and evaluate an individual’s ability to perform multiple motor tasks while walking.^[40]^ The FGA consists of 10 items, each rated on a 4-point scale from 0 to 3. A higher score signifies a higher level of functional walking ability. The highest score is 30 points. Measurements were taken 3 times in total, and the average value was recorded.^[41]^ It shows very high reliability in stroke patients with a test-retest ICC = .95.^[42]^

#### 2.3.3. 10-Meter walk test

The 10-Meter Walk test (10MWT) was conducted to measure the subject’s walking speed. 10MWT does not require special equipment and can easily evaluate walking speed in a short period of time.^[43]^ This method involves walking a total straight line distance of 14 meters at maximum speed and measuring the time required to walk a distance of 10 meters in the middle, excluding 2 meters at each end, while considering acceleration and deceleration times. Measurements were performed 3 times by a physical therapist with over 3 years of clinical experience, and the average value was reported. The reliability of the 10MWT for stroke patients was *r* = .89–1.00 for intra-rater reliability and *r* = .87 for inter-rater reliability.^[44]^

#### 2.3.4. 6-Minute walk test

The 6MWT was conducted in the hospital corridor. Each subject walked unaided back and forth along a 25-meter walkway for 6 minutes, with markers placed on the floor at intervals of 1 meter. The total distance walked was measured using the markers. The patients were allowed to self-adjust their walking speeds and rest times. The intra-rater reliability for the stroke patients was *r* = .99.^[39]^

### 2.4. Statistical analysis

The data collected in the study were analyzed using SPSS version 22.0 for Windows software (SPSS, Chicago, IL). For the general characteristics of the study subjects, descriptive statistics were used to calculate the mean and standard deviation. Homogeneity was assessed through the chi-square (χ^2^) test and independent *t*-test. The Kolmogorov–Smirnov test was conducted to assess normality. To compare groups over time, a 2-way repeated measures ANOVA was performed. The statistical significance level was set at α = .05.

If an interaction was observed, a one-way repeated measures ANOVA was conducted to compare within groups based on measurement time, and post hoc analysis was performed using the Bonferroni test. The level of statistical significance was set at α = .017. An independent *t*-test was conducted to compare between groups, followed by post hoc analysis using the Bonferroni test, with a statistical significance level of α = .025.

## 3. Results

### 3.1. General characteristics of the subjects

Flow chart for this study is shown in Figure 1. Table 2 provides a summary of clinical and demographic features of the sample (n = 34). The patients received sufficient explanation of the precautions during the training session before participating in the experiment. There were no significant (*P* > .05) differences in baseline characteristics between the 2 groups.

**Table 2:** General characteristics of the subjects.

Variable	e.g. (n = 17)	CG (n = 17)	*t/χ* ^*2*^	*P*
Gender				
Male	10 (58.8%)	12 (70.6%)	.515	.473
Female	7 (41.2%)	5 (29.4%)		
Paretic side				
Left side	9 (52.9%)	6 (35.3%)	1.074	.300
Right side	8 (47.1%)	11 (64.7%)		
Type of stroke				
Infarction	10 (41.2%)	8 (47.1%)	.472	.492
Hemorrhage	7 (58.8%)	9 (52.9%)		
Age (yr)	58.41 ± 9.29*	51.00 ± 13.44	−.915	.071
Time since onset (mo)	9.73 ± 2.87	11.63 ± 4.82	1.397	.172
Height (cm)	166.47 ± 9.35	167.98 ± 9.14	−.427	.637
Weight (kg)	69.14 ± 14.35	66.71 ± 11.49	.546	.589
FAC (score)	3.35 ± .49	3.53 ± .62	−.915	.367
MMSE-K (score)	27.53 ± 1.55	28.47 ± 1.28	1.933	.062

CG = control group, e.g. = experimental group, FAC = functional ambulation category, MMSE-K = Mini-Mental State Examination-Korean.

* Mean ± standard deviation.

### 3.2. All outcome measures for groups, time, and interaction

As a result of statistical analysis, the FGA, 10MWT, and 6MWT showed significant main effects of time, groups, and time × groups interaction effects (*P* < .05). However, the TUG showed significant main effects of time and time × groups interaction effects (*P* < .05), but did not show significant main effects of groups (*P* > .05) (Table 3).

**Table 3:** Statistical analysis results of all outcome measures for groups, time, and interaction.

Effects	Type III SS	df	Mean square	*F*	*P*
TUG (s)					
Time	194.611	1.571	123.840	302.916	.000*
Time × groups	5.667		3.606	8.821	.001*
Groups	34.081	1	34.081	1.130	.296
FGA (score)					
Time	102.176	1.462	69.912	87.672	.000*
Time × groups	14.529		9.941	12.467	.000*
Groups	84.794	1	84.794	4.283	.047*
10MWT (m/s)					
Time	.373	2	.187	218.472	.000*
Time × groups	.052		.026	30.623	.000*
Groups	.134	1	.134	5.023	.032*
6MWT (meter)					
Time	84,112.078	1.343	62,651.227	274.473	.000*
Time × groups	4546.196		3386.253	14.835	.000*
Groups	18,240.157	1	18,240.157	4.819	.036*

6MWT = 6-Minute Walk test, 10MWT = 10-Meter Walk test, FGA = functional gait assessment, TUG = timed up and go test.

* Statistical significance *P* <.05.

### 3.3. Comparison of all outcome measures within and between groups

As a result of the post hoc analysis, a significant difference was found between the post-test and the 2-week follow-up test compared to the pretest in all variables (*P* < .017) (Table 4). In between the 2 groups, there was a significant difference in the FGA, 10MWT, and 6MWT ratio in the pretest (*P* < .025) and the 2-week follow-up test (*P* < .025) (Table 4).

**Table 4:** Comparison of all outcome measures within and between groups.

Group	Pretest	Post-test	Follow-up (2 wk)	*F*	*P*
TUG (s)					
e.g. (n = 17)	14.76 ± 2.75†	11.83 ± 2.50‡	10.99 ± 2.53‡§	174.831	.000*
CG (n = 17)	15.27 ± 4.14	13.14 ± 3.51‡	12.62 ± 3.47‡§	128.231	.000*
*t*	.429	1.254	1.581		
*P*	.671	.219	.124		
FGA (score)					
e.g. (n = 17)	23.65 ± 2.71	26.41 ± 1.73‡	26.71 ± 1.86‡	57.004	.000*
CG (n = 17)	22.88 ± 3.22	24.18 ± 2.92‡	24.24 ± 3.03‡	31.442	.000*
*t*	−.749	−2.713	−2.863		
*P*	.459	.011**	.007**		
10MWT (m/s)					
e.g. (n = 17)	.72 ± .06	.91 ± .09‡	.87 ± .08‡§	139.978	.000*
CG (n = 17)	.71 ± .11	.80 ± .11‡	.78 ± .12‡§	81.296	.000*
*t*	.133	−3.314	−2.700		
*P*	.757	.002**	.011**		
6MWT (m)					
e.g. (n = 17)	276.71 ± 25.86	349.71 ± 38.83‡	352.94 ± 40.39‡	147.799	.000*
CG (n = 17)	268.24 ± 34.00	317.94 ± 39.17‡	312.94 ± 41.04‡	149.627	.000*
*t*	−8.18	−2.374	−2.864		
*P*	.420	.024**	.007**		

6MWT = 6-Minute Walk test, 10MWT = 10-Meter Walk test, CG = control group, EG = experimental group, FGA = functional gait assessment, TUG = timed up and go test.

† Mean ± standard deviation.

‡ *P* <.017 as compared to pretest (intragroup comparison).

§ *P* <.017 as compared to post-test (intragroup comparison).

* *P* <.017.

** *P* <.025.

**Figure 6.**
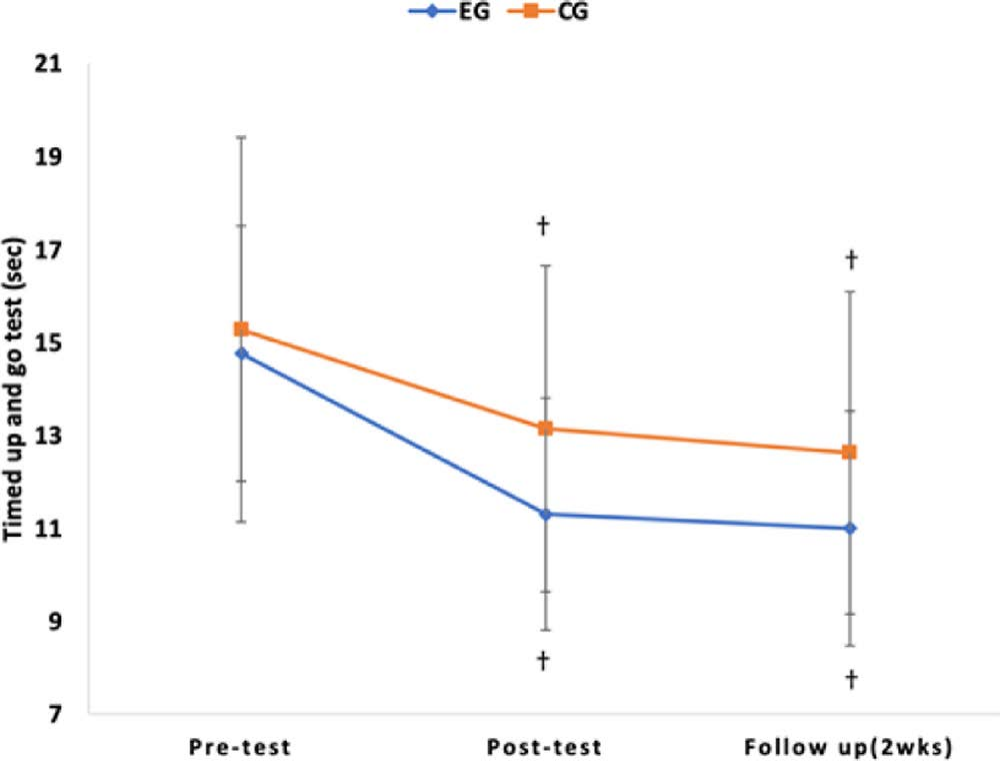
Comparison of the outcome measures within groups and change value of between groups in TUG. †*P* < .017 as compared to pretest (intragroup comparison). TUG = timed up and go.

**Figure 7.**
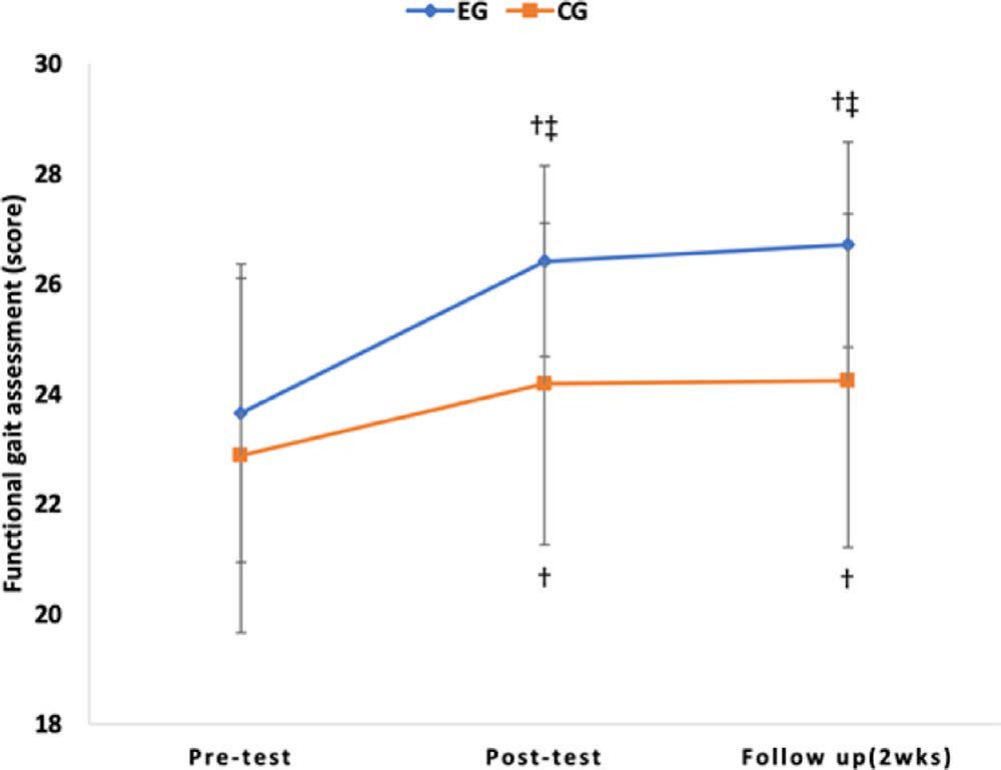
Comparison of the outcome measures within groups and change value of between groups in FGA. †*P* < .017 as compared to pretest (intragroup comparison). ‡*P* < .025 as compared to pretest (intergroup comparison). FGA = functional gait assessment.

**Figure 8.**
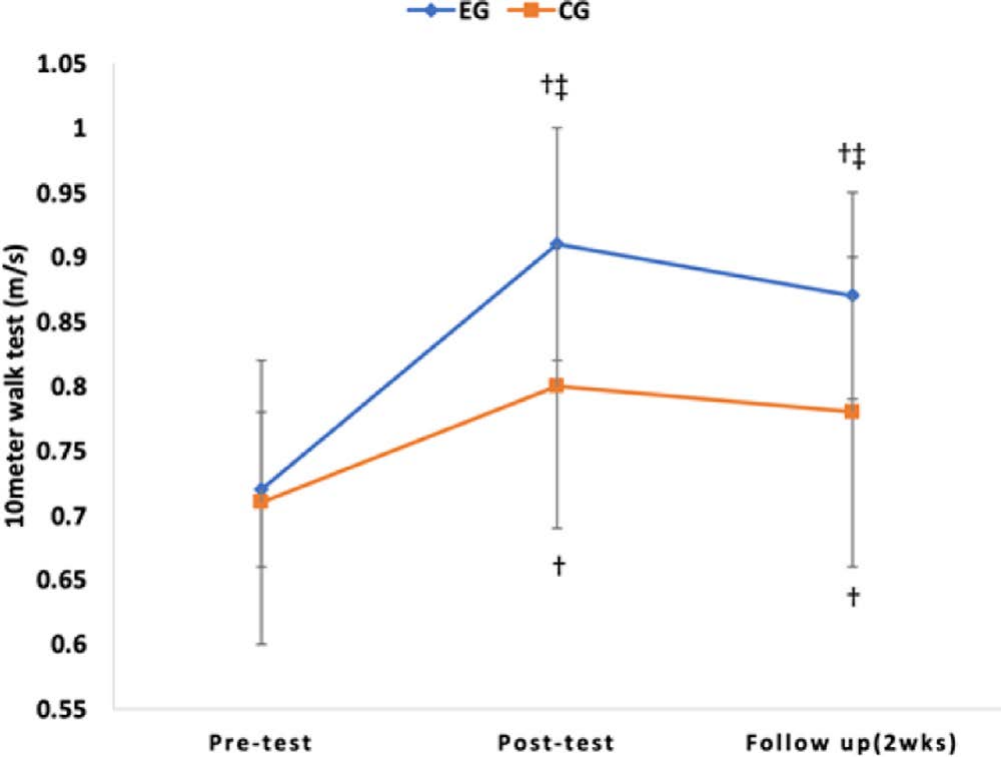
Comparison of the outcome measures within groups and change value of between groups in 10MWT. †*P* < .017 as compared to pretest (intragroup comparison). ‡*P* < .025 as compared to pretest (intergroup comparison). 10MWT = 10-meter walk test.

**Figure 9.**
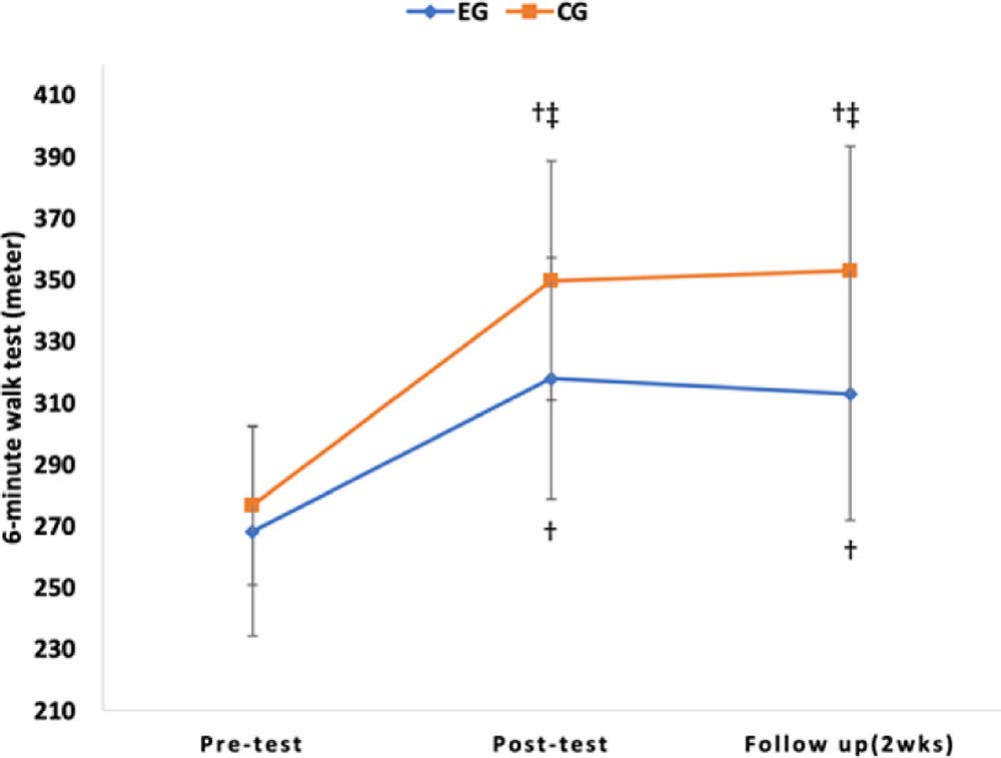
Comparison of the outcome measures within groups and change value of between groups in 6MWT. †*P* < .017 as compared to pretest (intragroup comparison). ‡*P* < .025 as compared to pretest (intergroup comparison). 6MWT = 6-minute walk test.

As a result of post hoc analysis, there was a significant decrease in TUG over time in the experimental group and control group (*P* < .017), but there was no significant difference in comparison between groups (*P* > .025) (Table 4). There was a significant increase in 10MWT, 6MWT, and FGA over time in the experimental and control groups (*P* < .017), and in the comparison between groups, there was a significant difference in the experimental group (*P* < .025) (Table 4) (Figs. 6, 7, 8, and 9).

## 4. Discussion

This study aimed to determine the effect of task-oriented interval treadmill training applied with high-intensity interval training on walking ability in chronic stroke patients. The experimental group underwent task-oriented treadmill training applied high-intensity interval training, and the control group underwent high-intensity interval treadmill training, 3 times a week for 30 minutes for 4 weeks. pretest and post-test for walking ability included TUG, 10MWT, 6MWT, and FGA. Additionally, to confirm the learning effect of the training, a follow-up test was performed 2 weeks after post-test.

In this study, a TUG was conducted to assess the change in dynamic balance. TUG is classified at the body functions level as an interpretation of balance in the International Classification of functioning, Disability, and Health (ICF) model,^[45]^ but detailed evaluation items are classified as activity level because they appear to be closely related to daily tasks.^[46]^ TUG comparison revealed significant differences over time in both trials, indicating an interaction effect between time and group. However, there was no significant difference between the experimental and control groups. The reason why there was no difference between the groups in TUG performance ability in this study is that the TUG test includes several elements including sitting-to-stand, walking upright, turning, and sitting-to-sitting movements. Task-oriented training specifically targeted obstacle passage during walking, but it did not address training related to the sitting-to-stand part of TUG, so it is thought that there was no difference between the 2 groups. The post-test results of the experimental group in the TUG test recorded 11.83 seconds, which was a 19.9% decrease, and showed a result higher than the minimal detectable change of 2.9 seconds in the TUG test. On the other hand, the post-test results of the control group recorded 13.14 seconds, which was a 13.9% decrease. Both groups exhibited a significant difference in the follow-up test, confirming the learning effect over time.

Ivar et al reported a significant difference in the TUG following 4 weeks of high-intensity aerobic training in chronic stroke patients.^[47]^ Additionally, Jeong and Koo reported significant improvements in dynamic balance ability during treadmill training with obstacle-crossing compared to general treadmill training in stroke patients.^[27]^ This is also consistent with the results of this study. In this study, it is believed that task-oriented obstacle-crossing training applied to the affected and unaffected sides, combined with high-intensity repetitive treadmill training, led to improved outcomes in dynamic balance tests such as TUG by necessitating continuous loss and recovery of balance capacity. According to a study by Said et al, crossing obstacles with one leg requires more swing time, while the supporting leg needs more stance time.^[48]^ This can contribute to balance training aimed at enhancing balance abilities. A study by Chou et al found that training obstacle-crossing with the affected legs of stroke patients increased muscle activity in hip flexor muscles.^[49]^ This supports the findings of this study, indicating that such training is effective in improving balance.

10MWT was performed to evaluate walking speed, and 6MWT was performed to assess walking endurance. As a result of this study, a significant difference was observed over time. There was also an interaction effect between time and group, along with significant differences between groups. Both the experimental group and the control group exhibited a significant difference between the pretest and the follow-up test, confirming the learning effect. The results of this study are consistent with previous studies that reported that high-intensity training in chronic stroke patients improved walking speed and walking endurance.^[18,36,47,50]^ Brauer et al reported that 8 weeks of high-intensity treadmill training increased walking endurance, walking speed, and aerobic capacity and had learning effects in stroke patients.^[19]^ These positive effects should be incorporated into self-management programs for stroke patients. Munari et al reported that high-intensity treadmill training improved walking ability, aerobic capacity, and walking efficiency more than low-intensity treadmill training.^[17]^ Schema theory can be used to explain the increase in walking speed, improvement of walking endurance, and learning effects of this study.^[51]^ Schmid et al stated that individuals acquire a generalized set of rules that can be applied to various situations.^[51]^ They particularly emphasized that generalized exercise programs consist of rules that establish spatial and temporal patterns of muscle activity required for executing a specific movement, which they referred to as a recall schema. As a result of this study, it is believed that repetitive training increased walking speed and endurance by learning a series of regular rules like a recall schema. In order to achieve good results when performing task-oriented training, it is necessary to select special and meaningful tasks for patients.^[52,53]^ Oh et al suggested that incorporating ample visual feedback is essential for regulating speed and maintaining target speed during treadmill training to enhance prefrontal cortex activity.^[54]^ As in this study, obstacle-climbing task training is a process of continuously repeating loss and recovery of balance in response to external disturbances. Despite being a chronic stroke patient, maintaining balance against external disturbances was thought to have a positive effect on walking speed and walking endurance through efforts to correct errors through spatiotemporal attention.^[55]^

Therefore, task-oriented training with high-intensity exercises seems to have a positive impact on brain plasticity and the recovery of activity levels, even in chronic stroke patients. In addition, as a result of the 10MWT post-test in this study, the control group increased by 0.09 m/s (12.7%) to 0.80 m/s, and the experimental group increased by 0.19 m/s (26.4%) to 0.91 m/s. This is a result of surpassing the Minimal Clinical Important Difference = 0.16 m/s for walking speed^[56]^ and 0.8 m/s, a threshold that indicates community walking ability.^[51]^ It is inferred that the experimental group’s likelihood of community walking can be anticipated. Fulk et al stated that stroke patients should be able to walk more than 288 meters without rest as a minimum requirement for successful community walking.^[57]^ For stroke patients with slow walking speed, walking about 300 meters without rest will be a very difficult and challenging task. The results of this study increased by 26.4% to 349.71 meters in the experimental group. This improvement substantially exceeds the minimal detectable change (MDC) of 36 meters reported for chronic stroke patients.^[39]^ The follow-up test also indicated more improvement compared to the control group. It is believed that the likelihood of community walking by the experimental group subjects can be predicted.

In this study, we compared FGA to assess walking performance. As a result of this study, significant differences were observed over time. Additionally, an interaction effect between time and groups was identified, along with significant differences between groups. Compared to previous studies, a study by Jung et al^[58]^ reported significant differences in FGA during obstacle-crossing walking training on various surfaces in stroke patients. These differences were attributed to the training’s effectiveness in providing real challenges. In addition, a study by Youn and Oh also reported the effect of repetitive training tailored to the environment.^[59]^ This type of training promotes voluntary muscle activity when task-oriented walking training is applied to stroke patients. A study by Leroux reported a greater improvement in gait function when partially reflected in the community environment by conducting walking training in places with various obstacles and stairs than in general walking training.^[60]^

In this study, the experimental group and the control group showed a significant difference. The application of practical task-oriented training, such as obstacle-crossing in the experimental group, caused posture sway through COM shifting. This led to an improvement in balance ability as a result of anticipatory postural adjustment and reactive postural adjustment. Therefore, it is believed that better results were achieved in the walking tasks by adjusting the walking speed and rotating the head in both horizontal and vertical directions.

The FGA is structured to enable the evaluation of complex walking situations while simultaneously assessing the performance of tasks related to walking and balancing abilities.^[40]^ Other previous studies have reported that cognitive ability and response to different environments are required to engage in other activities while walking.^[54,61]^ Therefore, in this study, meaningful tasks and continuous environmental changes are thought to have had a positive effect on walking in various environments. In addition, other activities during the walking process lead to the improvement of the activity of the prefrontal cortex and cognitive ability.^[61]^ Oh et al stated that the prefrontal cortex is activated during walking. However, when walking is combined with other tasks, the supplementary motor area is activated to adapt to different environments.^[54]^ The study reported an increase in activity in both the prefrontal cortex and the supplementary motor area as a result of treadmill training. According to Hubbard et al., performing a meaningful task leads to the reorganization of the cerebral cortex, thereby increasing brain plasticity.^[62]^ In addition, a review study by Takeuchi and Izumi^[63]^ reported that meaningful and repetitive high-intensity specific task training is important for stroke patients to recover motor function and neuroplasticity. Therefore, task-oriented training with high-intensity exercises is believed to have a positive impact on brain plasticity, enabling chronic stroke patients to walk in diverse environments.

The limitations of this study are as follows. Because the intervention period was short, there were variables that did not show statistically significant differences between the experimental and control groups. Summarizing the research results, it was possible to infer the possibility of walking in the community by setting up an intervention program similar to the actual environment. However, the actual transfer effect could not be confirmed because the community’s walking ability was not closely evaluated. In addition, the study is limited by the small sample size of 34 subjects, making it is difficult to generalize the findings to all stroke patients, and Spatiotemporal gait variables were not measured.

## 5. Conclusion

As a result of this study, both the task-oriented treadmill training group and the high-intensity interval training group showed a positive impact on walking ability over time. Additionally, interaction and learning effects with time and group were also confirmed. In particular, the task-oriented treadmill training with high-intensity showed significant differences in walking speed, walking endurance, and walking performance compared to the high-intensity interval treadmill training group. As a result of this study, it is believed that even chronic stroke patients can experience a positive impact on their walking ability and engagement in community activities by engaging in meaningful tasks at high-intensity activity levels. This approach is also believed to facilitate a quicker recovery for inpatients to resume their daily lives.

## Acknowledgments

We thank our colleagues and the reviewers who provided constructive criticism on drafts of this essay.

## Author contributions

**Conceptualization:** Kyu-Ryeong Kim.

**Data curation:** Kyu-Ryeong Kim.

**Formal analysis:** Kyu-Ryeong Kim.

**Investigation:** Ye-Ji Kim.

**Methodology:** Ye-Ji Kim.

**Project administration:** Ye-Ji Kim.

**Resources:** Ye-Ji Kim.

**Software:** Ye-Ji Kim, Myoung-Kwon Kim.

**Supervision:** Myoung-Kwon Kim.

**Validation:** Myoung-Kwon Kim.

**Visualization:** Myoung-Kwon Kim.
